# BALF editome profiling reveals A-to-I RNA editing associated with severity and complications of *Mycoplasma pneumoniae* pneumonia in children

**DOI:** 10.1128/msphere.01012-24

**Published:** 2025-02-25

**Authors:** Yun-Yun Jin, Yun Guo, Su-Wan Xiong, Na Zhang, Jian-Huan Chen, Feng Liu

**Affiliations:** 1 Laboratory of Genomic and Precision Medicine, Wuxi School of Medicine, Jiangnan University, Wuxi, Jiangsu, China; 2 Joint Primate Research Center for Chronic Diseases, Institute of Zoology of Guangdong Academy of Science, Jiangnan University, Wuxi, Jiangsu, China; 3 MOE Medical Basic Research Innovation Center for Gut Microbiota and Chronic Diseases, Wuxi School of Medicine, Jiangnan University, Wuxi, Jiangsu, China; 4 Department of Respiratory Medicine & Clinical Allergy Center, Affiliated Children’s Hospital of Jiangnan University (Wuxi Children’s Hospital), Wuxi, Jiangsu, China; 5 Department of Ophthalmology, Affiliated Hospital of Jiangnan University, Wuxi, Jiangsu, China; 6 Department of Respiratory Medicine, Children’s Hospital of Nanjing Medical University, Nanjing, Jiangsu, China; University of Wisconsin-Madison, Madison, Wisconsin, USA

**Keywords:** *Mycoplasma pneumoniae *pneumonia, A-to-I RNA editome, ADAR, epitranscriptomic, immune response

## Abstract

*Mycoplasma pneumoniae* is an important human respiratory pathogen that causes mild-to-moderate community-acquired *M. pneumoniae* pneumonia (MPP), particularly in children. RNA editing plays a vital role in pathogen infection and host immune response, but it remains largely unknown how it could be involved in the epigenetic regulation of host response to *M. pneumoniae* infection. In the present study, we performed an epitranscriptomic analysis of adenosine to inosine (A-to-I) editing in 39 bronchoalveolar lavage fluid (BALF) samples from the severe side (SS) and the opposite side (OS) of patients with MPP. Our editome profiling identified 87 differential RNA editing (DRE) events in 50 genes, especially missense editing events that recoded C-C motif chemokine receptor-like 2 (*CCRL2*, p.K147R) and cyclin I (*CCNI*, p.R75G). The expression of adenosine deaminase acting on RNA (*ADAR*) significantly increased on SS compared to OS and positively correlated with the average RNA editing level and individual DRE events. Meanwhile, functional enrichment analysis showed that DRE was observed in genes primarily associated with the negative regulation of innate immune response, type I interferon production, and cytokine production. Further comparison of SS between complicated MPP (CMPP) and non-complicated MPP (NCMPP) revealed RNA editing events associated with MPP complications, with a higher *ADAR* expression in CMPP than NCMPP. By identifying DRE events as biomarkers associated with MPP severity and complications, our editome profiling provides new insight into the potential role played by A-to-I RNA editing in modulating the host’s immune system during *M. pneumoniae* infection.

**IMPORTANCE:**

Our research investigates how *Mycoplasma pneumoniae*, a common respiratory pathogen, influences how our cells modify their genetic instructions. By studying RNA editing changes in bronchoalveolar lavage fluid from patients with *M. pneumoniae* pneumonia, we aim to investigate how *M. pneumoniae* infection alters epigenetics and contributes to the disease severity and complications. Understanding such epigenetic alterations not only sheds light on the mechanisms underlying *M. pneumoniae* infection but also holds potential implications for developing better diagnostic tools and therapies. Ultimately, this work may facilitate the design of more targeted treatments to alleviate the impact of respiratory infections caused by the pathogen. Our findings may also offer broader insights into how microbial infections reshape immune processes, highlighting the importance of RNA editing in host-pathogen interactions.

## INTRODUCTION

*Mycoplasma pneumoniae* is a unique pathogen that could infect humans and animals to cause walking pneumonia and other respiratory disorders (1). In addition to its small size and genome, the *Mycoplasma* genus is distinguished from other prokaryotes by the lack of cell wall structure, which makes it insensitive to beta-lactam antibiotics (2, 3). Although most of its infections are asymptomatic, it could cause community-acquired pneumonia, especially in children. Up to 40% or more community-acquired pneumonia in children was *M. pneumoniae* pneumonia (MPP), with up to 18% of cases requiring hospitalization (4–6). Globally, MPP is recognized to have epidemic peaks that recur every 3–7 years, with each outbreak lasting 1–2 years (4, 7–9). Nevertheless, the underlying mechanisms of MPP severity remain largely unelucidated, which is essential for preventing and treating the disease.

Epigenetics plays a substantial role in the immune response to infection. RNA editing catalyzed by adenosine deaminase acting on RNA (ADAR) converts adenosine to inosine (A-to-I) in RNA and could potentially influence mRNA splicing, stability, translation efficiency, and encoded protein function (10). RNA editing is a critical component of the host immune response to viral and intracellular bacterial infections (11–13), whereas its role in other microbial infections needs to be further explored. Although many *M. pneumoniae* infections remain asymptomatic (14), recent studies suggest that, in some cases, such infections can induce significant immune responses and have long-term effects on host health (5, 15). The difference in molecular landscape and underlying mechanisms related to such different outcomes remains elusive. A-to-I RNA editing may play a critical role in modulating the host’s immune responses to infections (16). Therefore, the involvement of A-to-I RNA editing in host immune response to MPP could be of particular importance.

Our current study comprehensively investigated A-to-I RNA editome in bronchoalveolar lavage fluid (BALF) samples collected from MPP children, providing new insight into its potential role in host immune response and the development of diagnostic markers.

## MATERIALS AND METHODS

### Clinical data and sample collection

The inclusion criteria for children with MPP were based on expert consensus on laboratory diagnostics and clinical practice of MPP in children in China as previously described (17). In brief, the inclusion criteria for children with MPP were as follows: (i) diagnosed with community-acquired pneumonia; (ii) confirmed to be positive with *M. pneumoniae* infection by qPCR of nasopharyngeal aspirates; and (iii) unilateral pneumonia on chest imaging, as this study specifically focused on cases of MPP characterized by unilateral pneumonia. Patients were excluded for the following: (i) tested positive for other pathogens in a range of diagnostic assays, including BALF cultures, viral antigen detection, and immunological assays such as the purified protein derivative test, interferon-gamma release assays, and T-cell spot tests on any samples from throat swabs, nasopharyngeal aspirates, sputum, BALF, or blood; (ii) with pre-existing health issues like immunodeficiency, chronic illnesses, or cardiac conditions, and those on immunosuppressive medication; (iii) without guardian consent for participation.

A total of 21 children diagnosed with MPP who, despite treatment with macrolide antibiotics, had persistent fever or incomplete resolution of pulmonary symptoms on radiographic imaging were subjected to fiberoptic bronchoscopy and chest imaging. MPP in children often appears as unilateral lesions on chest imaging, which could indicate pneumonia, lung consolidation, atelectasis, or pleural effusion. The sides presenting these characteristics were used as the severe side (SS), while the other side was used as the opposite side (OS). According to a previous study (18), we further stratified the SS group into complicated MPP (CMPP) and non-complicated MPP (NCMPP) subgroups. CMPP was defined by the presence of both local complications, such as parapneumonic effusion, empyema, necrotizing pneumonia, and lung abscess, and systemic complications, including bacteremia, metastatic infection, multi-organ failure, acute respiratory distress syndrome, disseminated intravascular coagulation, and, in rare cases, death. Cases of MPP without such complications were classified as NCMPP.

BALF was sampled from the most severely affected lung lobe on SS and a comparable lobe on OS during fiberoptic bronchoscopy after general anesthesia by administering a combination of intravenous and inhalational agents. When the bronchoscope was lodged in the target bronchus, 37°C normal saline was injected through the working channel (1 mL/kg/time, ≤20 mL/time, and the total amount ≤5–10 mL/kg). Then, BALF was obtained by negative pressure aspiration at 100–200 mmHg. Chest X-ray or CT was used to determine the affected lobes, which allowed us to divide them into SS and OS. We first performed lavage on the OS to obtain BALF, followed by lavage on the SS using the same method. The collected BALF samples were promptly processed and stored at −80°C for subsequent testing. All samples were subjected to bulk RNA sequencing, ensuring consistent handling across all cases.

### RNA-seq reads processing

The raw RNA-seq reads from our previous study (PRJCA015679) (5) were included and reanalyzed using a pipeline described in our previous study, and FASTQC was used to assess the quality of RNA-Seq reads for quality control (QC) (19). After QC, 21 SS and 18 OS samples were included in our subsequent analysis. Reads that passed QC were aligned to the human genome reference (UCSC hg38) with RNA STAR version 2.7.0e (20). Following alignment, the generated BAM files were filtrated using SAMtools version 1.9 (21) to remove multi-mapped reads or optical duplicates. Subsequently, base quality score recalibration was performed using the Genome Analysis Toolkit (GATK) version 4.1.3, following its guidelines (22).

### RNA editing analysis

Single nucleotide variants (SNVs) were called from the BAM files utilizing VarScan version 2.4.4 (23), followed by annotation using the Ensembl Variant Effect Predictor (VEP) (24). Only A-to-G SNVs on the coding strands or T-to-C on the non-coding fulfilling these criteria were retained: a base quality score of 25 or above, a total sequencing depth of ten or more, an alternative allele depth of at least 2, and an alternative allele frequency (AAF) of at least 1%. SNVs were excluded if they met any of these conditions unless recorded as RNA editing sites in the REDIportal V2.0 database (25): (i) located in homopolymeric regions of five or more nucleotides or simple sequence repeats; (ii) located in mitochondrial DNA; (iii) located within six nucleotides of a splice junction; (iv) located within one nucleotide of an insertion or deletion (Indel); (v) located within 4% proximity to the read starts or ends; (vi) listed as known variants in the dbSNP database Build 142; (vii) observed in over 90% of samples with an AAF of either 100% or between 40% and 60%.

### Sorting intolerant from tolerant (SIFT) prediction

The SIFT web server (http://sift-dna.org) was utilized to predict the impacts of amino acid alterations in proteins arising from missense A-to-I RNA editing (26).

### Gene expression quantification

The Bioconductor package Rsubread version 2.8.2 was used to calculate the pseudo counts and normalized gene expression levels expressed as transcripts per million (TPM) for RNA expression (27).

### Function enrichment analysis

Gene ontology (GO) and Kyoko Encyclopedia of Genes and Genomes (KEGG) analyses were conducted utilizing the DAVID Bioinformatics Resources online platform (https://david.ncifcrf.gov/tools.jsp) and the Enrichr tool (https://maayanlab.cloud/Enrichr/) (28). The significance cut-off of a false discovery rate (FDR) <0.05.

### RNA-binding protein (RBP) binding site prediction

For a better understanding of the potential functional impact of RNA editing, the RBPmap online tool (http://rbpmap.technion.ac.il) was employed to predict potential RBP binding sites that coincide with the locations of RNA editing (29).

### DNA and RNA extraction

Genomic DNA and total RNA were extracted from collected BALF samples following standard protocols. For DNA extraction, FastPure Cell/Tissue DNA Isolation Mini Kit-BOX1 (Vazyme, Cat: DC102-01) was used following the manufacturer’s instructions. Total RNA was extracted using the RNAsimple Total RNA Extraction Kit (Tiangen, REF: DP419), following the manufacturer’s instructions. The quality and quantity of the extracted DNA and RNA were assessed using a Nanodrop spectrophotometer (Thermo Fisher Scientific) and agarose gel electrophoresis.

Reverse transcription PCR (RT-PCR) was performed with 1 µg of total RNA. First-strand cDNA synthesis was carried out using the HiScriptIII RT SuperMix for qPCR (+gDNA wiper) cDNA synthesis kit (Vazyme, R323), following the manufacturer’s protocol.

### PCR amplification for RNA editing site validation

To confirm the presence of RNA editing at specific loci, we designed primers flanking the target editing sites (Table S1). The resulting DNA and cDNA were then subjected to PCR amplification using the primers designed for the specific target regions.

PCR was carried out using the Phanta Max Super-Fidelity DNA Polymerase (Vazyme, P505) according to the manufacturer’s protocol. The PCR products were analyzed using agarose gel electrophoresis.

The PCR products were subsequently subjected to Sanger sequencing to confirm the presence of A-to-I RNA editing events at the target sites.

### Diagnostic performance analysis

To evaluate the diagnostic performance for predicting MPP severity and complications, the editing levels of selected RNA editing events were subjected to the receiver operating characteristic (ROC) curve analysis and used as independent variables to construct classifiers in logistic regression implemented in SPSS (version 22). The status of MPP severity and complications was used as the dependent variables. The values of the area under the ROC curve (AUC) were calculated.

### Statistical analysis

In statistical analysis, RNA editing levels between SS versus OS and NCMPP versus CMPP were compared using the general linearized model (GLM) method and likelihood ratio test to calculate the empirical *P* values (*P*_GLM_). Categorical data were subjected to analysis via Fisher’s exact test. The correlation between levels of RNA editing and gene expression was analyzed using the Pearson and Spearman rank correlation methods to obtain the correlation coefficients (*r*) and *P* values.

## RESULTS

### Clinical characteristics of 21 MPP children

A total of 21 children who underwent fiberoptic bronchoscopy with MPP were prospectively enrolled in the Respiratory Department of Children’s Hospital of Nanjing Medical University from January to December 2021. Characteristics of these subjects, including the age, hospitalization duration, absolute neutrophil count, C-reactive protein (CRP), alanine aminotransferase (ALT), aspartate aminotransferase (AST), lactate dehydrogenase (LDH) and creatine kinase-MB (CK-MB) levels, and counts of platelets and white blood cells, are summarized in Table 1.

**TABLE 1:** The clinical characteristics of 21 MPP children^*a*^

Age (years)	Hospitalization duration (days)	WBC (×10^9^/L)	CRP (mg/L)	HB (g/L)	PLT (×10^9^/L)	ALT (U/L)	AST (U/L)	LDH (U/L)	CK-MB (U/L)
5	17	10.66	42	129	416	47	33	556	29
9.67	9	5.04	10	127	159	7	19	319	11
4	9	18.74	10	129	362	95	28	695	25
6.67	12	12.86	52	121	354	14	13	300	13
8.42	19	12.02	32	118	337	30	41	557	21
13.75	8	7.11	30	133	192	41	61	509	18
8.08	12	12.82	20	130	235	18	33	678	38
8.5	6	6.65	2.828	115	339	8	17	241	12
4.58	14	6.72	11	131	253	19	41	844	24
9.33	16	4.75	54	116	178	22	47	441	19
7	12	6.19	11	122	250	8	32	375	16
7	9	13.54	2.828	133	397	16	26	245	18
9.83	27	10.58	14	121	382	67	50	478	13
3.5	12	4.53	35	127	162	18	30	458	30
13.17	18	11.04	9	145	214	13	17	425	22
5.5	9	13.2	9	122	353	19	26	413	18
3.08	10	7.38	29	120	251	13	32	223	25
7.5	9	8.75	2.828	121	294	14	33	351	17
6	20	2.83	34	122	169	136	106	688	34
8.17	7	9.6	2.828	132	350	97	48	353	19
9.5	9	8.54	2.828	133	278	16	27	311	16

^*a*^ ALT, alanine aminotransferase; AST, aspartate aminotransferase; CK-MB, creatine kinase-MB; CRP, C-reactive protein; HB, hemoglobin; LDH, lactate dehydrogenase; PLT, platelet; WBC, white blood cell.

### A-to-I RNA editing in BALF from MPP children

**Fig 1:**
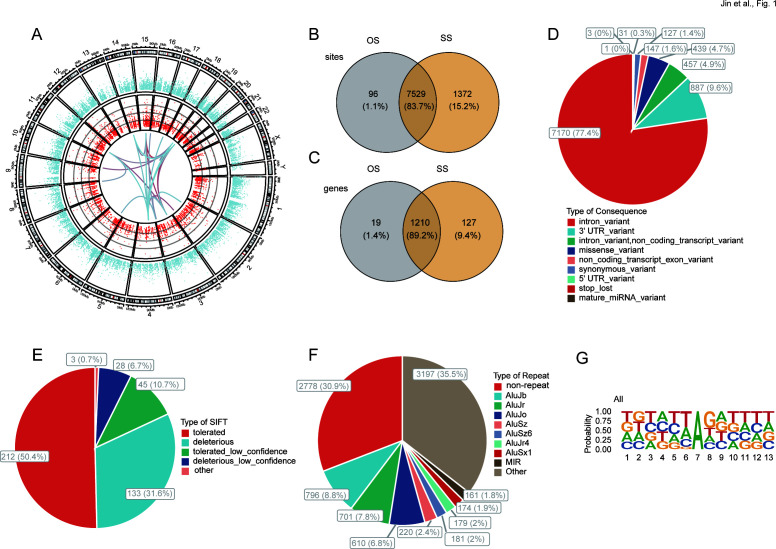
A-to-I RNA editing identified from BALF transcriptome of MPP children. (**A**) Circos plot of transcription gene expression (outer circle) and A-to-I RNA editing sites (inner circle) in the MPP children BALF. (**B and C**) The A-to-I RNA editing events (**B**) and genes (**C**) between OS and SS. (**D**) Functional types of variants resulted from A-to-I RNA editing. (**E**) About 38.3% of missense variants are predicted by SIFT to be possibly deleterious to the encoded proteins. (**F**) 33.6% of RNA editing events are in *Alu* repetitive elements. (**G**) The motif of sequence motif surrounding the A-to-I RNA editing sites. Six nucleotides upstream and downstream of the editing sites are shown.

In total, 8,997 A-to-I RNA editing events with high confidence were identified in 1,356 genes in BALF samples from all children studied (Fig. 1A). The editing levels of these events varied from 1% to 100% and were found throughout the human genome. Among these events, 7,529 (83.7%) were shared between OS and SS. In comparison, 96 (1.1%) and 1,372 (15.2%) were exclusively observed in OS and SS, respectively (Fig. 1B). As for edited genes, 1,210 (89.2%) were shared between OS and SS, with 19 (1.4%) and 127 (9.4%) genes being uniquely edited in OS and SS, respectively (Fig. 1C). The top three functional categories of these RNA editing events comprised 7,170 (77.4%) intronic, 887 (9.6%) 3′-untranslated region (3′ UTR), and 439 (4.7%) missense variants (Fig. 1D). SIFT predicted that 161 (38.3%) of the missense variants might influence the function of the encoded proteins (Fig. 1E). Additionally, 33.6% of these edited events were located in *Alu* repetitive elements (Fig. 1F). Examination of the sequences surrounding these candidate editing sites revealed a pattern with guanine suppressed at the −1 upstream base and preferred at one base downstream of the editing sites (positions indicated as 6 and 8 in Fig. 1G).

### Changes in BALF RNA editing associated with MPP severity

**Fig 2:**
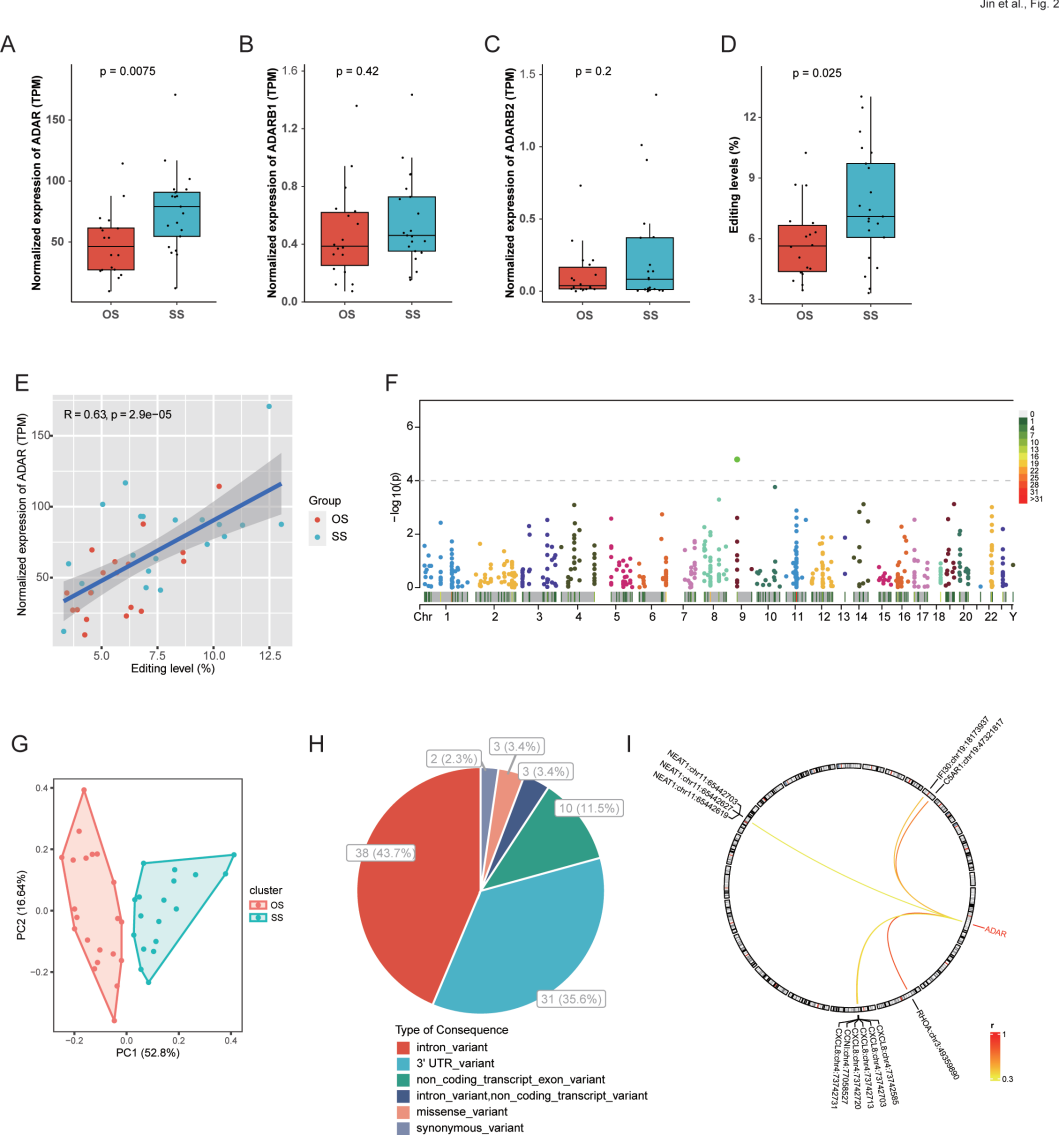
A-to-I RNA editing in OS and SS. (**A–C**) The mRNA expression of *ADAR* (**A**), *ADARB1* (**B**), and *ADARB2* (**C**). (**D**) The overall average editing level. (**E**) The correlation of overall average editing level and gene expression of *ADAR*. (**F**) Manhattan plot showing the distribution of DRE sites in the BALF transcriptome. (**G**) The PCA of the DRE events between OS and SS. (**H**) Functional types of DRE variants. (**I**) The correlation between *ADAR* and DRE sites ( Spearman correlation *r* > 0.3 and *P* < 0.05).

*ADAR* increased significantly in SS compared to OS (Fig. 2A), whereas *ADARB1* and *ADARB2* showed no significant changes (Fig. 2B and C). The transcriptome-wide average editing level significantly increased in SS compared to OS (Fig. 2D), positively correlated with the *ADAR* expression level (Fig. 2E).

Among these RNA editing events, 87 differential RNA editing (DRE) sites were observed in 50 genes (Fig. 2F; Table S2). Principal component analysis (PCA) using these DRE sites separated SS and OS samples well, with PC1 accounting for 52.8% of the total variation (Fig. 2G). The top three functional categories of these DRE sites included 38 (43.7%) intronic, 31 (35.6%) 3′ UTR, and 10 (11.5%) non-coding transcript exonic variants, with a notably increased proportion of 3′ UTR variants compared to that in the overall RNA editing variants (Fig. 2H). Further analysis revealed a subset of DRE events positively correlated with the *ADAR* expression level (Fig. 2I).

### MPP severity-associated BALF RNA editing was mainly involved in immune- and virus-related gene functions and pathways

**Fig 3:**
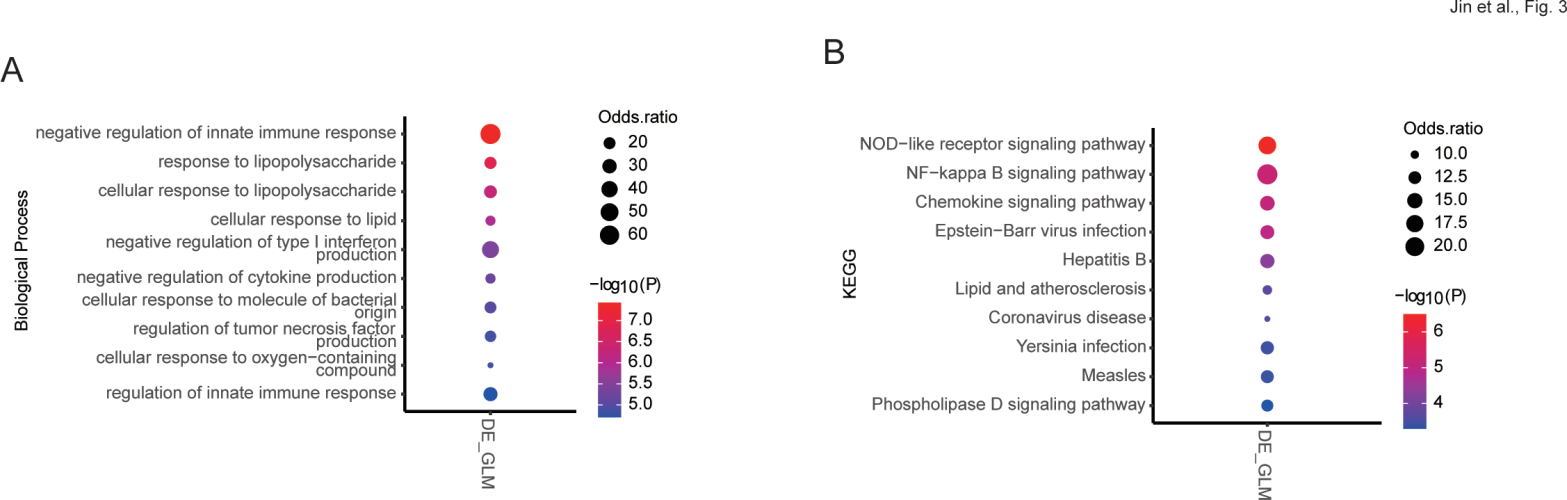
Enrichment of gene ontology and KEGG pathways in genes with DRE. The items are shown for (**A**) biological processes and (**B**) KEGG pathway enriched by genes differentially edited between OS and SS.

Gene function enrichment showed that the BALF DRE observed between SS and OS of MPP patients was mainly involved in immune and virus-related biological processes, such as the negative regulation of the innate immune response, type I interferon production, and cytokine production (Fig. 3A), and KEGG pathways, such as the NOD-like receptor signaling pathway, the NF-kappa B signaling pathway, the chemokine signaling pathway, Epstein-Barr virus infection, and hepatitis B (Fig. 3B).

### Proteins recoded by missense RNA editing associated with MPP severity

**Fig 4:**
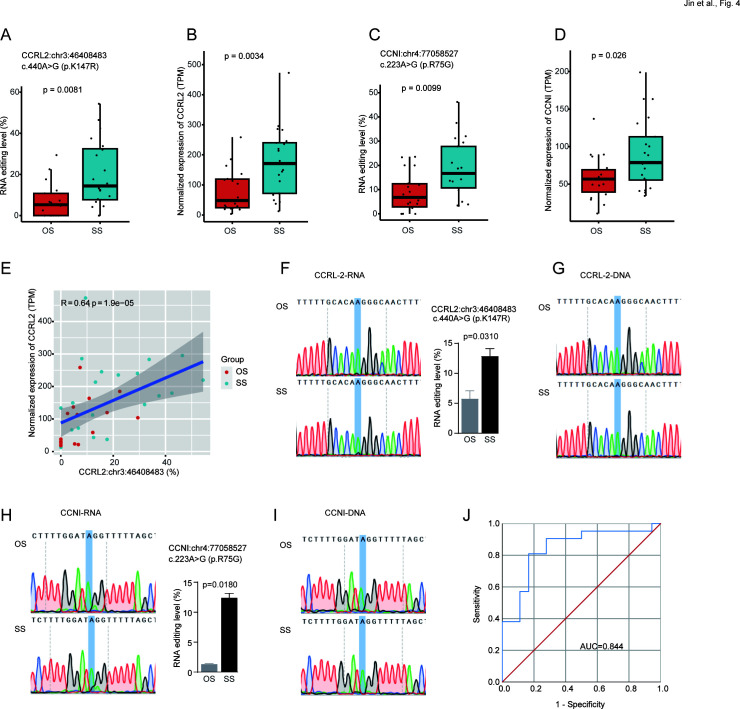
The two genes with missense DRE. (**A and B**) *CCRL2*:chr3:46408483 editing level (**A**) and gene expression level (**B**). (**C and D**) *CCNI:*chr4:77058527 editing level (**C**) and gene expression level (**D**). (**E**) The *Sperman* correlation of *CCRL2*:chr3:46408483 editing level and gene expression level. (**F and G**) The Sanger sequencing peaks of *CCRL2*:chr3:46408483 at the RNA (**F**) and DNA (**G**) levels. (**H and I**) The Sanger sequencing peaks of *CCNI:*chr4:77058527 at the RNA (**H**) and DNA (**I**) levels. (**J**) ROC curves are generated using a combination of *CCNI:*chr4:77058527 and *CCRL2*:chr3:46408483.

We subsequently investigated missense RNA editing associated with MPP severity, potentially recoding protein and influencing protein structure and stability (30). Two missense DRE events were observed in C-C motif chemokine receptor-like 2 (*CCRL2*, p.K147R) and cyclin I (*CCNI*, p.R75G). The editing levels (*CCRL2*:chr3:46408483 and *CCNI*:chr4:77058527) and gene expression levels were significantly increased in SS (Fig. 4A through D). Among them, *CCRL2* p.K147R was located in its trans-membrane domain, whereas *CCNI* p.R75G was predicted to be deleterious in its cyclin-like domain. In addition, a strong positive correlation with the gene expression was observed for the *CCRL2* missense editing (Fig. 4E) but not for the *CCNI* missense editing (data not shown).

Sanger sequencing was used to validate the presence of A-to-I RNA editing events at the target sites. The results showed that the *CCRL2*:chr3:46408483 and *CCNI:*chr4:77058527 variants were detected at the RNA level but not at the DNA level, and the editing levels were higher in SS than OS (Fig. 4F through I).

ROC curves were used to assess further the potential of *CCRL2*:chr3:46408483 and *CCNI*:chr4:77058527 as biomarkers for diagnosing MPP severity. The classifiers constructed by combining these two missense editing sites showed an AUC value of 0.844 (Fig. 4J). These results indicate that RNA editing of *CCRL2*:chr3:46408483 and *CCNI*:chr4:77058527 could serve as biomarkers for MPP severity prediction.

### MPP severity-associated 3′ UTR DRE in BALF might contribute to gene expression regulation

**Fig 5:**
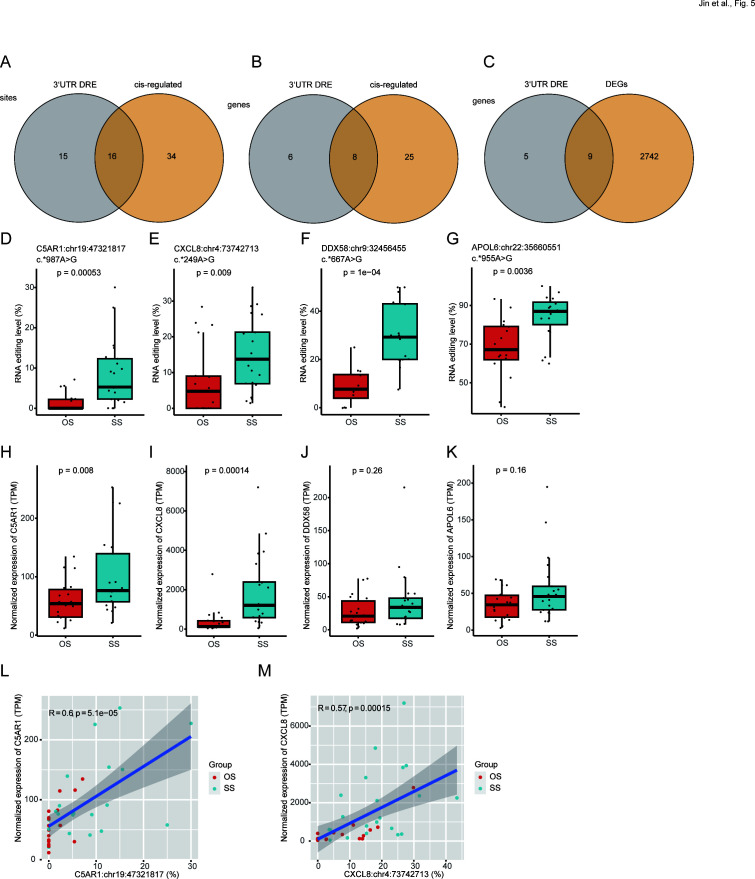
3′ UTR DRE with *cis*-regulatory effects. (**A and B**) Venn plot showed the shared DRE sites (**A**) and genes (**B**) between 3′ UTR and *cis*-regulated. (**C**) Venn plot showed the shared DRE genes between 3′ UTR and differentially expressed genes. (**D–K**) *C5AR1*:chr19:47321817, *CXCL8*:chr4:73742713, *DDX58*:chr9:32456455, and *APOL6*:chr22:35660551 A-to-I RNA editing level (**D–G**), and gene expression level (**H–K**). (**L and M**) Scatter plots showing *cis*-regulatory effects of *C5AR1*:chr19:47321817 (**L**) and *CXCL8*:chr4:73742713 A-to-I RNA editing on gene expression level (**M**).

We then focused on the potential *cis*-regulatory effects of 3′ UTR RNA editing. Our study identified 31 3′ UTR DRE events in 14 genes (Table S2), with 16 (51.6%) in 8 genes predicted to exert *cis*-regulation (Fig. 5A and B; Table S3). Furthermore, nine of these differentially edited genes (64.3%) exhibited differential gene expression (Fig. 5C; Table S4). The top 10 significantly differentially edited 3′ UTR variants ranked by *P* values are shown in Table 2. Notably, one of the most pronounced differential editing was found in the complement C5a receptor 1 (*C5AR1*), C-X-C motif chemokine ligand 8 (*CXCL8*), DExD/H-box helicase 58 (*DDX58*), and apolipoproteins L6 (*APOL6*) genes. All the RNA editing (*C5AR1*:chr19:47321817, *CXCL8*:chr4:73742713, *DDX58*:chr9:32456455, and *APOL6*:chr22:35660551) and edited gene expression levels except the expression of *DDX58* and *APOL6* significantly increased in SS (Fig. 5D through K). The mRNA expression and site editing levels of *C5AR1* and *CXCL8* genes also showed a significant *cis*-regulated association (Fig. 5L and M). The top 10 *cis*-regulated DRE events ranked by the *Spearman* correlation coefficient are shown in Table 3.

**TABLE 2:** The top 10 3′ UTR events with the most significant differential editing^*a*^

Event_id	NT change	Known site	*P*_GLM_	Correlation between RNA editing and gene expression		Average editing level (%)	
				*P*_Spearman_	*P*_Pearson_	OS	SS
DDX58:chr9:32456455	c.*667A > G	TRUE	1.61e−05	0.064	0.239	8.8	30.0
C5AR1:chr19:47321817	c.*987A > G	TRUE	5.28e−04	5.06e−05	3.41e−05	1.4	8.4
CXCL8:chr4:73742731	c.*267A > G	TRUE	8.12e−04	2.19e−07	1.51e−04	7.5	18.4
APOL6:chr22:35660551	c.*955A > G	TRUE	9.77e−04	0.037	0.088	68.0	84.1
ADGRE2:chr19:14734257	c.*1979A > G	TRUE	0.001	0.449	0.795	45.7	65.5
ERO1A:chr14:52643092	c.*478A > G	TRUE	0.001	0.155	0.073	20.1	38.5
DDX58:chr9:32456367	c.*755A > G	TRUE	0.002	0.359	0.998	31.4	58.3
SGTB:chr5:65667685	c.*2561A > G	TRUE	0.003	0.012	0.130	1.3	4.5
GBP4:chr1:89183779	c.*1475A > G	TRUE	0.004	0.291	0.021	64.7	40.6
APOL6:chr22:35660685	c.*1089A > G	TRUE	0.004	0.614	0.709	33.0	51.1

^*a*^ *P*_GLM_: the differential editing level between OS and SS. Known site: the RNA editing site has been found in the database. TRUE: known.

**TABLE 3:** The top 10 *cis*-regulated events with the most significant differential editing

Site_id	Consequence	NT change	AA change	*P*_GLM_	*P*_Spearman_	Average editing level (%)	
						OS	SS
CXCL8:chr4:73742731	3′ UTR	c.*267A > G	No	8.12e−04	2.19e−07	7.5	18.4
CCRL2:chr3:46408483	Missense	c.440A > G	p.K147R	0.005	1.87e−05	7.4	20.1
TNFAIP3:chr6:137878768	Synonymous	c.1323A > G	p.E441	0.002	3.11e−05	0.6	2.8
C5AR1:chr19:47321817	3′ UTR	c.*987A > G	No	5.28e−04	5.06e−05	1.4	8.4
PTK2B:chr8:27425209	Intron	c.551 + 2826A > G	No	0.019	7.11e−05	7.5	15.8
MTND2P28:chr1:630318	Exon	n.679A > G	No	0.003	1.87e−04	58.1	73.0
ITGAX:chr16:31359000	Intron	c.431–700A > G	No	0.005	5.67e−04	11.7	22.6
SGK1:chr6:134170115	3′ UTR	c.*153A > G	No	0.008	7.46e−04	4.7	12.3
LINC-PINT:chr7:131100079	Intron	n.1524 + 7641A > G	No	0.020	0.001	20.3	34.7
EGR3:chr8:22689300	3′ UTR	c.*1173A > G	No	0.016	0.001	2.5	6.1

### Hyper-edited genes with MPP severity-associated RNA editing

Our findings also underscored the significant role of genes possessing multiple RNA editing sites in the BALF of SS. A total of 38 genes, which constituted 100% of the differentially edited genes, were identified to harbor two or more editing sites, as detailed in Table 4. The top three were LYN proto-oncogene, Src family tyrosine kinase (*LYN*) with 259 editing sites, dedicator of cytokinesis 4 (*DOCK4*) with 202 editing sites, and *AC099489.1* with 145 editing sites. All the top 10 genes showed increased expression, especially lipopolysaccharide-induced TNF factor (*LITAF*), and *LYN* expression significantly increased in SS (*P* = 0.001 and *P* = 0.008, respectively) (Table 5).

**TABLE 4:** Enrichment of differential A-to-I RNA editing genes with multiple editing sites

	Edited_sites		*P* value^*a*^
	1	Two or more	
Differentially_edited			<0.001
No	592 (45%)	726 (55%)	
Yes	0 (0%)	38 (100%)	

^*a*^ Pearson’s chi-squared test.

**TABLE 5:** Top 10 genes with the largest counts of A-to-I RNA editing events^*a*^

Gene	Number of detected editing events	With significantly different A-to-I RNA editing level	Gene expression level (TPM)		*P* value^*b*^
			OS	SS	
LYN	259	Yes	78.8	176.6	0.008
DOCK4	202	Yes	17.4	33	0.017
AC099489.1	145	Yes	23.8	36.6	0.068
LITAF	141	Yes	113.9	238.5	0.001
PSTPIP2	114	Yes	29.7	65.9	0.036
GK	112	Yes	130.4	216.3	0.115
ACSL1	106	Yes	87.2	177	0.019
FNDC3B	103	Yes	59.8	112.4	0.015
AZIN1-AS1	102	Yes	8.5	23.1	0.056
RBM47	102	Yes	25.6	36.1	0.058

^*a*^ Transcripts per million (TPM) represents a relative expression level.

^*b*^ Shows the difference *P*-values for gene expression between OS and SS calculated using edgeR.

### RNA editing in BALF associated with MPP severity might influence RBP binding activity

**Fig 6:**
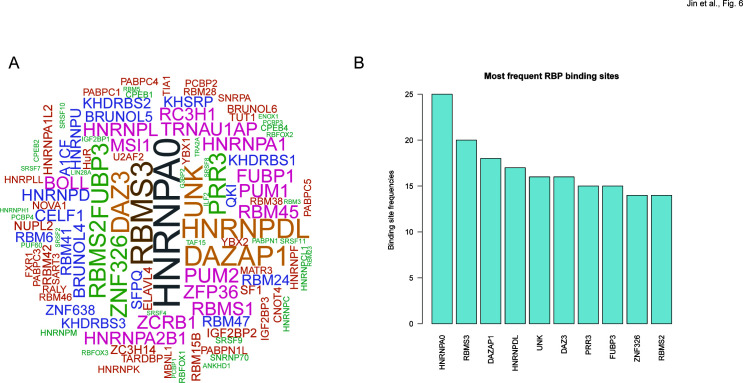
RNA editing in BALF associated with MPP severity might influence RBP binding activity. (**A**) Word cloud showing RBPs with binding sites overlapping MPP severity-associated DREs. (**B**) Top 10 RBPs with the most frequent binding sites overlapping MPP severity-associated DREs.

To assess the possible impact of RNA editing on the binding of RBPs, the RBPmap database was used to predict the binding sites of RBPs that overlapped with the DRE sites. Figure 6 shows the top RBPs sorted by the frequency of their overlapping DRE sites, with the top three being heterogeneous nuclear ribonucleoprotein A0 (HNRNPA0), RNA binding motif single-stranded interacting protein 3 (RBMS3), and DAZ-associated protein 1 (DAZAP1).

### A-to-I RNA editing in BLAF associated with complications in MPP

**Fig 7:**
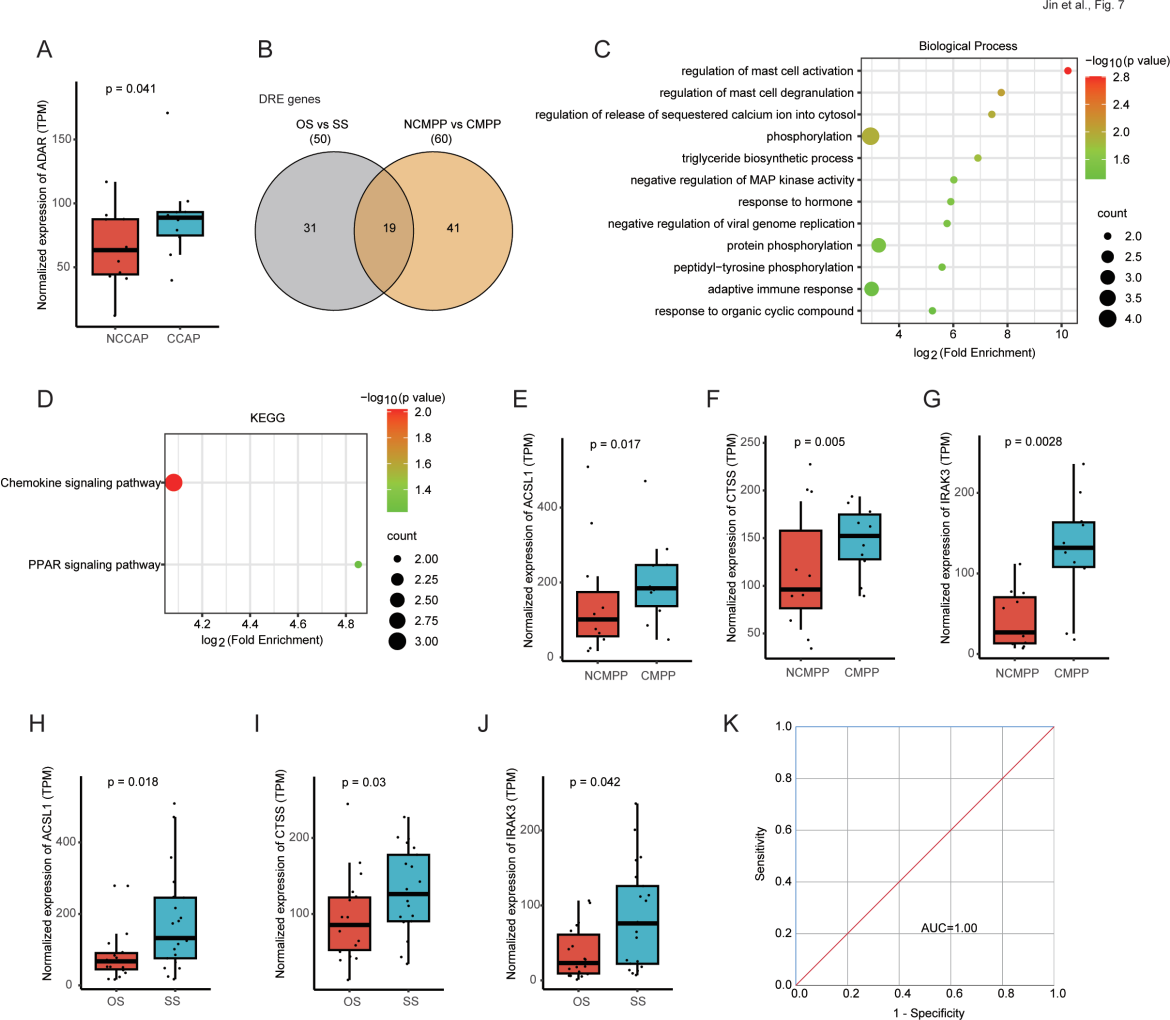
A-to-I RNA editing in BLAF associated with complications in MPP. (**A**) The expression of *ADAR*. (**B**) The shared DRE genes in OS versus SS and NCMPP versus CMPP. (**C and D**) The items are shown for (**C**) biological processes and (**D**) KEGG pathway enriched in 19 shared genes with Fold Enrichment. (**E–J**) The expression of *ACSL1* (**E and H**), *CTSS* (**F and I**), and *IRAK3* (**G and J**) in CMPP and SS compared with their controls, respectively. (**K**) ROC curves are generated using a combination of *ACSL1* (chr4:184824208/184823799), *CTSS* (chr1:150732881/150732178/150732032), and *IRAK3* (chr12:66194142/66194056/66194058).

Due to the significant RNA editing alterations observed in SS, we sought to further delineate the association of complications with RNA editing in SS. Consequently, we stratified the SS group into NCMPP and CMPP subgroups. Upon analysis, our study discovered that there was also a significant alteration in the expression of *ADAR* between NCMPP and CMPP (Fig. 7A). Eighty-two DRE events were identified in 60 genes between NCMPP and CMPP (Table S5), among which 19 genes were also differentially edited between OS and SS (Fig. 7B). Gene function enrichment showed that these 19 shared DRE genes were mainly involved in mast cell and immune-related biological processes such as regulation of mast cell activation, degranulation, and adaptive immune response (Fig. 7C), and KEGG pathways such as chemokine signaling pathway, PPAR signaling pathway (Fig. 7D). Among these 19 shared genes, three including acyl-CoA synthetase long-chain family member 1 (*ACSL1*), cathepsin S (*CTSS*), and interleukin 1 receptor-associated kinase 3 (*IRAK3*) genes showed significantly increased expression, which were positively associated with MPP severity and complications (Fig. 7E through J).

ROC curves were used to further assess the potential application of *ACSL1* (chr4:184824208/184823799), *CTSS* (chr1:150732881/150732178/150732032), and *IRAK3* (chr12:66194142/66194056/66194058) editing sites as biomarkers for diagnosing MPP complications. The classifier constructed by combining these editing sites showed an AUC value of 1.00 (Fig. 7K). These results indicate that the editing levels of these sites could be potential biomarkers for predicting MPP complications.

## DISCUSSION

Before our current study, there was limited understanding of the underlying epigenetic alterations and mechanisms of RNA editing related to MPP severity. Through a systematic investigation of A-to-I RNA editing in BALF samples of MPP, our study underlined a potentially significant involvement of A-to-I RNA editing in modulating the immune response related to MPP severity.

Our findings showed that *ADAR* expression, along with the mean level of A-to-I RNA editing, increased significantly with MPP severity. This suggests that upregulated RNA editing activity, emphasizing the interplay between RNA editing machinery and host defense mechanisms, is a crucial component of the innate immune response against MPP of varying severity. ADAR, an enzyme renowned for its function in post-transcriptional RNA modification (31, 32), demonstrates a significant upregulation in response to pathogenic invasion. The promoted expression of ADAR might enhance the host’s resistance to pathogenic infections by reshaping the transcriptome for anti-pathogenic responses, possibly by changing the secondary structure of RNA and regulating the immunogenicity of both cellular self-RNA and pathogenetic RNAs (33–37). Our findings suggest the vital role of ADAR in modulating the host’s innate and adaptive immune responses to counteract pathogenic infections effectively.

Missense RNA editing alters the amino acid sequences, potentially enhancing protein diversity or influencing protein structure, stability, and functions (30). Our study observed significantly upregulated missense RNA editing of two genes, *CCRL2* (chr3:46408483) and *CCNI* (chr4:77058527), in SS. *CCRL2* encodes a chemokine receptor-like protein, a seven-transmembrane domain receptor located within a CC chemokine receptor cluster on human chromosome 9 (38–40). Chemokine signaling is crucial for guiding immune cell migration to inflammatory sites (41–44). CCRL2 is highly expressed in primary neutrophils and monocytes, with an increased expression upon neutrophil stimulation and during monocyte differentiation into macrophages (44). Additionally, CCRL2 regulates immune responses in inflammation-related diseases, such as experimental autoimmune encephalitis and inflammatory arthritis (45–48). The correlations between levels of *CCRL2* RNA editing and gene expression might be critical for better understanding the potential effects of RNA editing in gene expression regulation involved in responses to MPP of varying severity, warranting further investigation into the functional roles of RNA editing in MPP.

The RNA editing in 3′-UTR can result in alterations in the targeting, maturation of microRNA, and interaction with RBPs (49–51). Among the genes with 3′-UTR DRE, C5AR1 is the receptor of C5A, a key pro-inflammatory mediator, an anaphylatoxin (52), and a component of the complement pathway involved in innate and adaptive immune responses (53). It could promote inflammation and/or repair processes in ischemic stroke (54). Additionally, COVID-19 patients with critical conditions exhibited extensive activation of the C5a-C5aR1 pathway (55). The *CXCL8* gene encodes a member of the CXC chemokine family and a major mediator of pro-inflammatory responses (56, 57). It serves as a chemoattractant, directing neutrophils to the site of infection (57–59). Products from bacteria and viruses could trigger CXCL8 expression during inflammation (56, 58). Excessive CXCL8 might lead to pulmonary inflammation (60, 61) and cystic fibrosis (62). Therefore, our findings of such 3′ UTR RNA editing changes associated with MPP severity could align with the role of these genes in anti-infection defense and immune regulation.

The detection of RNA editing using high throughput sequencing might be influenced not only by the editing level but also by the gene expression level. RNA editing could be easier to detect in genes with high expression levels. Conversely, low expression levels may affect the detection of RNA editing. This variability poses a challenge when interpreting DRE results across various conditions or treatments. Future studies should also consider employing additional methodologies, such as control samples with known editing profiles and target sequencing, to validate the observed RNA editing sites more rigorously.

In our analysis of RBPs, many DRE sites overlapped with binding sites of HNRNPA0, a type I interferon (IFN)-repressed host factor in HIV-1-infected cells (63). Knockdown hnRNPA0 increased the viral particle yield, consequently enhancing viral infectivity. Conversely, overexpression of hnRNPA0 notably reduced the overall viral mRNA and protein levels (63). Additionally, RBMS3 belongs to the c-myc gene single-strand binding protein family. RBMS3 is a post-transcription regulator involved in immune evasion by regulating PD-L1, and reduced RBMS3 levels enhance auranofin-induced anti-tumor T-cell responses (64). Our findings thus suggested that DRE in the BALF from children with MPP of varying severity could potentially influence RBP binding activity to the RNA of DRE genes and in turn regulate the host immune response.

Furthermore, our study revealed that *ADAR* expression also significantly increased with MPP complications. The observed enrichment of genes with DRE in immune response, particularly the involvement of mast cells and adaptive immunity, underscores the complexity of the host’s response to infection, suggesting a possible link between RNA editing and MPP complications. *ACSL1*, *CTSS*, and *IRAK3* were identified as key target genes, and the combination of multiple DRE sites in these genes showed a promising diagnostic performance for MPP complications. Such findings further emphasized that RNA editing could be worth investigating in identifying epigenetic biomarkers and therapeutic targets related to MPP severity and complications, and further research is needed to validate these findings comprehensively.

Our study has several limitations. First, while we performed BALF culture to identify potential co-infections, bacterial isolates were excluded as co-infections, which led to the unavailability of antibiotic susceptibility data. This exclusion could limit the broader understanding of how bacterial pathogens might have interacted with *M. pneumoniae* in MPP, especially given the patients’ prior failure of macrolide therapy. Additionally, the methods used in our current study limited cytological analysis of BALF. Although standard in many histopathological examinations, HE staining did not allow for blood cell differentiation or detailed analysis of major cell types such as neutrophils, lymphocytes, or macrophages. A more detailed cytological assessment is needed to explore the inflammatory or immune cell profiles in response to *M. pneumoniae* infection.

In conclusion, our current study thoroughly profiled BALF A-to-I RNA editome and revealed dynamic changes related to the severity and complications of MPP.

## Data Availability

The data set associated with this study (BioProject accession PRJCA015679) is publicly available in the CNCB-NGDC (China National Center for Bioinformation) repository (https://ngdc.cncb.ac.cn/). The permanent URL for the data set is https://ngdc.cncb.ac.cn/bioproject/browse/PRJCA015679.

## SUPPLEMENTAL MATERIAL

Captions msphere.01012-24-s0001.docx Captions for supplemental tables.

Supplemental Tables msphere.01012-24-s0002.xlsx Tables S1-S5.

ASM does not own the copyrights to Supplemental Material that may be linked to, or accessed through, an article. The authors have granted ASM a non-exclusive, world-wide license to publish the Supplemental Material files. Please contact the corresponding author directly for reuse.
