# Supplementary material for: BALF editome profiling reveals A-to-I RNA editing associated with severity and complications of Mycoplasma pneumoniae pneumonia in children
Source: mSphere. 2025 Feb 25;10(3):e01012-24. doi: 10.1128/msphere.01012-24 (PMC11934315; doi:10.1128/msphere.01012-24)
Supplement: Captions — for supplemental tables. [file msphere.01012-24-s0001.docx]

# Supplementary Figure Legends

**Table S1. The primers for PCR amplification and Sanger sequencing.**

**Table S2. DRE sites associated with MPP severity.**

**Table S3. The shared genes and sites of 3′ UTR DRE and *cis*-regulated.**

**Table S4. The shared genes of 3′ UTR DRE and differentially expressed genes.**

**Table S5. DRE sites associated with complications in MPP.**
